# Characteristics of Cardiac Abnormalities in Pediatric Patients With Acute COVID-19

**DOI:** 10.7759/cureus.36093

**Published:** 2023-03-13

**Authors:** Daniel Pasternack, Rakesh K Singh, Prashant K Minocha, Jon S Farkas, Prema Ramaswamy, Donna Better, Sourabh Verma, Colin K Phoon

**Affiliations:** 1 Pediatric Cardiology, Hassenfeld Children's Hospital at New York University (NYU) Langone, New York, USA; 2 Pediatrics, New York University Grossman School of Medicine, New York, USA; 3 Pediatrics, New York City (NYC) Health + Hospitals/Bellevue, New York, USA; 4 Pediatric Cardiology, Maimonides Medical Center, Brooklyn, USA; 5 Pediatric Cardiology, New York University (NYU) Langone Hospital - Long Island, Mineola, USA; 6 Pediatrics, New York University Long Island School of Medicine, Mineola, USA; 7 Neonatology, Hassenfeld Children's Hospital at New York University (NYU) Langone, New York, USA

**Keywords:** myocarditis, electrocardiogram, pediatrics, ventricular dysfunction, covid-19

## Abstract

Introduction

Coronavirus disease 2019 (COVID-19) is known to cause cardiac abnormalities in adults. Cardiac abnormalities are well-described in multisystem inflammatory syndrome in children, but effects in children with acute COVID-19 are less understood. In this multicenter study, we assessed the cardiac effects of acute COVID-19 among hospitalized children (<21 years) admitted to three large healthcare systems in New York City.

Methods

We performed a retrospective observational study. We examined electrocardiograms, echocardiograms, troponin, or B-type natriuretic peptides.

Results

Of 317 admitted patients, 131 (41%) underwent cardiac testing with 56 (43%) demonstrating cardiac abnormalities. Electrocardiogram abnormalities were the most common (46/117 patients (39%)), including repolarization abnormalities and QT prolongation. Elevated troponin occurred in 14/77 (18%) patients and B-type natriuretic peptide in 8/39 (21%) patients. Ventricular dysfunction was identified in 5/27 (19%) patients with an echocardiogram, all of whom had elevated troponin. Ventricular dysfunction resolved by first outpatient follow-up.

Conclusion

Electrocardiogram and troponin can assist clinicians in identifying children at risk for cardiac injury in acute COVID-19.

## Introduction

While coronavirus disease 2019 (COVID-19), caused by the severe acute respiratory syndrome coronavirus 2 (SARS-CoV-2), primarily targets the respiratory system, it is known to injure other tissues as well, including the heart [1,2]. Examples of cardiac abnormalities in adult patients range from mild derangement in markers of cardiac injury or nonspecific electrocardiogram changes to pericardial effusion, systolic ventricular dysfunction, myocardial infarction, and myocarditis [3-5]. Specific electrocardiogram changes include prolongation of the QT interval and repolarization abnormalities [3,6]. Tachyarrhythmias were also reported [4]. Patients with evidence of cardiac involvement are at increased risk of death [3,7].

The etiology of cardiac injury in COVID-19 has not yet been elucidated; a combination of direct viral infection and local vascular disease may be responsible in the setting of certain modifiable and non-modifiable risk factors, such as gender, race, and preexisting conditions [8].

Children with COVID-19 generally have favorable outcomes compared to adults, although this is not always the case and some children experience severe disease occasionally resulting in death [1,9]. Cardiac changes occur in up to 20% of pediatric patients [2,10]. Like adults, children demonstrate nonspecific electrocardiogram changes, conduction disturbances (including heart block), tachyarrhythmias, and left ventricular dysfunction [10-12]. Subclinical myocardial changes consistent with myocarditis have also been seen in routine screening of college athletes [13]. Children with comorbidities, especially obesity and asthma, appear to be at increased risk of severe disease [14].

Due to the favorable outcomes in children, the majority of COVID-19 research has focused on adults. Fewer studies have assessed the cardiac effects of COVID-19 in children, with most of this small subset focused on the effects of the COVID-19-related multisystem inflammatory syndrome in children (MIS-C) [10]. In this multicenter study, we describe patterns of cardiac abnormalities in pediatric patients with acute COVID-19.

## Materials and methods

Study population

We performed a retrospective observational study across three large hospital systems in New York City. All patients aged 21 years and younger hospitalized between March 1, 2020, and February 28, 2021, with a positive polymerase chain reaction test for SARS-CoV-2, were included, regardless of the indication for testing or admission. To limit the study population strictly to patients with acute COVID-19, patients diagnosed with MIS-C were excluded [15]. For patients admitted more than once during the study period with a positive SARS-CoV-2 polymerase chain reaction, only the first hospitalization was included. For patients transferred between hospitals, only the site of the highest level of care was included. Notably, no study participant had received a vaccine against SARS-CoV-2.

Data collection and definitions

This study was approved by the Institutional Review Boards of New York University Grossman School of Medicine (s20-00428) and Maimonides Medical Center (2020-12-21), and the authors conducted the research in accordance with the principles stated in the Declaration of Helsinki. Through chart review, we identified demographic information, including the area deprivation index (ADI), a decile based on census block groups that measure socioeconomic disadvantage within each state [16,17]. Past medical history and presenting symptoms were also extracted.

After collecting patients who satisfied our inclusion criteria without meeting the criteria for exclusion, we identified patients who underwent cardiac testing, defined as having at least one of the following performed during admission: an electrocardiogram, echocardiogram, and measurement of serum B-type natriuretic peptide or troponin. Cardiac abnormality was defined as the presence of an abnormality in one or more of these four tests. We considered an electrocardiogram abnormal if a significant anomaly was identified, including repolarization abnormalities (such as ST segment and/or T wave abnormalities), corrected QT interval prolongation, atrial enlargement, ventricular hypertrophy, axis deviation, heart block, low voltage, or premature ventricular contractions. The corrected QT interval was considered prolonged if greater than 470 milliseconds for males and 480 milliseconds for females [18]. Echocardiograms were considered abnormal if ventricular dysfunction, atrioventricular valve dysfunction, ventricular hypertrophy, pericardial effusion, or increased pulmonary artery pressure were identified. When possible, electrocardiograms and echocardiograms were compared to studies from prior to the admission to exclude preexisting abnormalities.

Cardiac magnetic resonance imaging (MRI) data were reviewed when available. Other test results, including complete blood counts, electrolytes, inflammatory markers (including erythrocyte sedimentation rate, C-reactive protein, procalcitonin, and ferritin), coagulation markers (including fibrinogen and D-dimer), and chest radiographs, were collected. Hospital site, level of care, length of stay, and required therapies with specific attention to vasoactive medications, medications used for antiviral purposes at the time of patient care (including remdesivir, azithromycin, hydroxychloroquine, or zinc), and those used for anti-inflammatory purposes (including systemic steroids, tocilizumab, or anakinra) were also recorded. Lastly, for each patient found to have a cardiac abnormality, follow-up cardiac testing results were recorded.

Statistical analyses

We compared available characteristics between patients based on whether or not they underwent cardiac testing, as well as between tested patients based on whether or not they were found to have a cardiac injury. Differences in the characteristics between groups were analyzed using a Fisher’s exact test for categorical data or a Wilcoxon rank-sum test for continuous data. Differences in the proportions of patients screened for cardiac abnormalities over different time periods during the study were analyzed using the Pearson correlation coefficient. Statistical significance was defined as a p value of <0.05. All data were analyzed using Statistical Package for the Social Sciences (SPSS) for Windows version 25 (IBM SPSS Statistics, Armonk, NY, USA).

## Results

Patterns in screening for cardiac abnormalities

**Figure 1:**
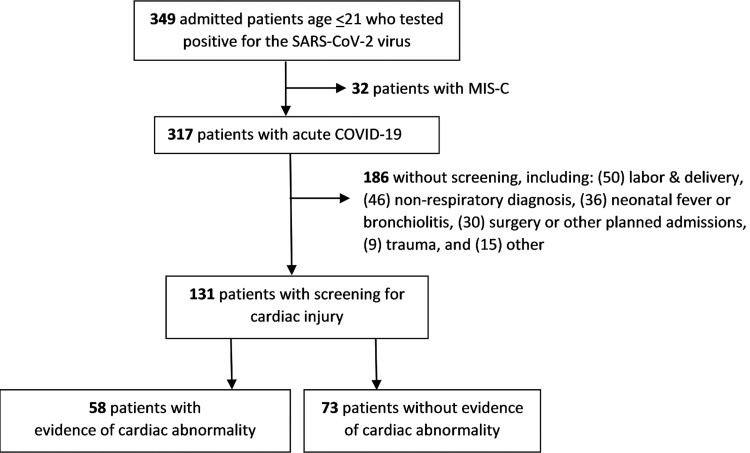
Study cohort SARS-CoV-2: severe acute respiratory syndrome coronavirus 2, MIS-C: multisystem inflammatory syndrome in children, COVID-19: coronavirus disease 2019

Three hundred forty-nine admitted patients tested positive for SARS-CoV-2, while 32 of these patients were diagnosed with MIS-C. Of the remaining 317 patients, 131 (41%) underwent screening for cardiac abnormalities (Figure 1). Patients who did not undergo cardiac screening tended to be admitted for infections outside of the respiratory tract (such as cellulitis or gastroenteritis), noninfectious conditions, and planned surgery or procedure. In addition, patients admitted with bronchiolitis or neonatal fever were often not screened.

Screened patients were more likely to be older, have preexisting medical conditions, have symptoms consistent with COVID-19, require respiratory support for a longer duration of time, and have longer lengths of stay (Table 1).

**Table 1:** Patient characteristics by cardiac testing status ADI: area deprivation index

Characteristic	Cardiac testing (n = 131)	No cardiac testing (n = 186)	p
Age, years (interquartile range)	17 (3-19)	10 (1-19)	0.042
ADI, decile (interquartile range)	3 (2-5)	3 (2-5)	0.12
Existing medical condition (%)	76 (58%)	89 (48%)	0.087
Symptoms on presentation (%)	106 (81%)	101 (54%)	<0.0001
Duration of illness, days (interquartile range)	2 (1-5)	1 (1-2)	<0.0001
Hospital			0.45
1	40 (30%)	46 (25%)	
2	12 (9%)	29 (15%)	
3	27 (21%)	34 (18%)	
4	39 (30%)	57 (31%)	
5	13 (10%)	20 (11%)	
Level of care			<0.0001
Acute	75 (58%)	121 (65%)	
Intensive	49 (37%)	15 (8%)	
Obstetrics	2 (1%)	50 (27%)	
Other (surgical and psychiatric)	5 (4%)	0 (0%)	
Respiratory support requirement (%)	54 (41%)	10 (5%)	<0.0001
Length of stay, days (interquartile range)	4 (2-11)	2 (1-3)	<0.0001
Primary diagnosis			<0.0001
Cardiac	4 (3%)	0 (0%)	
Gastrointestinal	8 (6%)	8 (4%)	
Infection (not respiratory)	9 (7%)	11 (6%)	
Labor and delivery	1 (1%)	50 (27%)	
Neonatal fever	7 (5%)	33 (18%)	
Neurologic	8 (6%)	16 (9%)	
Respiratory	60 (46%)	16 (9%)	
Surgery	7 (5%)	31 (16%)	
Trauma	8 (6%)	10 (5%)	
Other	19 (15%)	11 (6%)	

**Figure 2:**
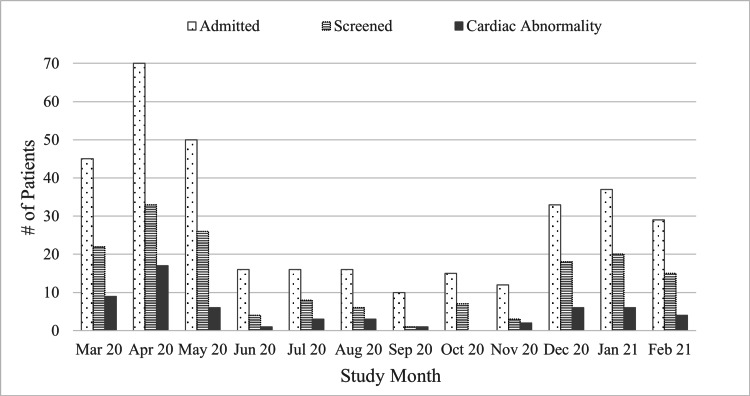
Patterns in cardiac injury and screening over time

The proportion of patients screened for cardiac abnormalities did not significantly change over the study period (r = -0.577, p = 0.063) (Figure 2).

Abnormal cardiac testing

Of the 131 patients screened for cardiac abnormalities, 56 (43%) had an abnormal cardiac test (Tables 2-4). The most common electrocardiogram abnormality was repolarization abnormality, seen in 29 (25%) patients with electrocardiograms (Table 2). Prolonged corrected QT interval was seen in nine (8%) patients who exhibited a median corrected QT interval of 496 milliseconds (interquartile range: 482-531 milliseconds).

**Table 2:** Electrocardiogram abnormalities 1: QTc prolonged if >470 msec in males and >480 msec in females

Electrocardiogram (n = 117)	Abnormality
Any abnormality	49 (42%)
Repolarization abnormality	29 (25%)
ST segment abnormality	14 (12%)
T wave abnormality	19 (16%)
Prolonged QT interval^1^	9 (8%)
Median QTc, milliseconds (interquartile range)	496 (482-531)
Left axis deviation	4 (3%)
Right axis deviation	3 (2%)
Left atrial enlargement	5 (4%)
Right atrial enlargement	1 (1%)
Left ventricular hypertrophy	5 (4%)
Right ventricular hypertrophy	4 (3%)
Low voltage	2 (2%)
Prolonged PR interval	2 (2%)
Heart block (second or third degree)	0 (0%)
Premature ventricular contraction	1 (1%)

**Table 3:** Laboratory abnormalities on admission 1: normal troponin < 0.040 ng/mL, 2: normal B-type natriuretic peptide < 100 pg/mL

Laboratory measurements	Results
Serum troponin (n = 77)	
Number elevated	14 (18%)
Value of elevated results (on admission), ng/mL^1 ^(median, interquartile range, range)	0.065, 0.021-0.35, 0.010-3.24
Serum B-type natriuretic peptide (n = 39)	
Number elevated	8 (21%)
Value of elevated results (on admission), pg/mL^2 ^(median, interquartile range, range)	269, 117-312, 106-601

**Table 4:** Echocardiogram abnormalities

Echocardiograms (n = 27)	Abnormality
Any abnormality	7 (26%)
Left ventricular dysfunction	4 (15%)
Right ventricular dysfunction	1 (4%)
Atrioventricular valve regurgitation (>mild)	1 (4%)
Elevated right ventricular pressure	1 (4%)
Pericardial effusion	1 (4%)
Coronary artery abnormality	0 (0%)

Serum troponin was elevated in 14 (18%) patients, and B-type natriuretic peptide was elevated in eight (21%) patients (Table 3). Seven of 27 (26%) echocardiograms were abnormal, including five with ventricular dysfunction, one with a pericardial effusion, and one with elevated right ventricular pressure. No coronary artery abnormalities were identified (Table 4).

**Figure 3:**
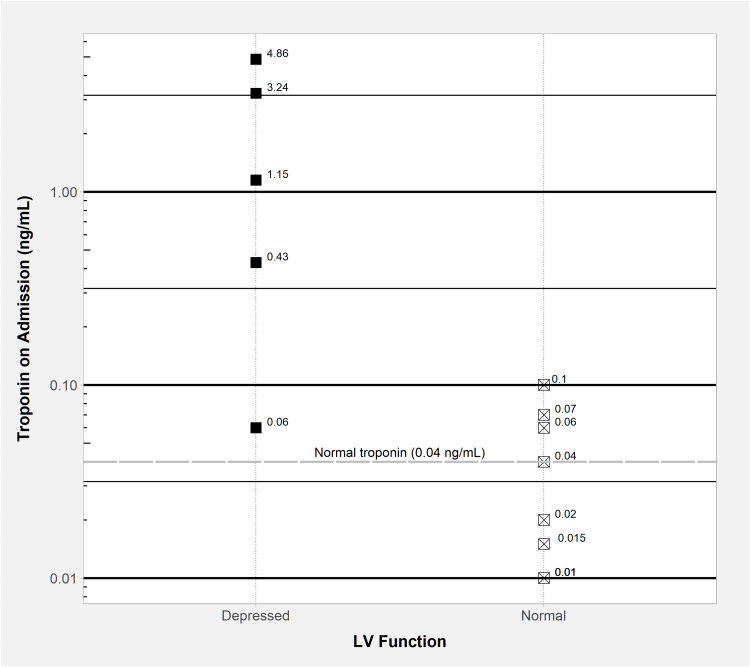
Difference in serum troponin on admission between patients with and without ventricular dysfunction by echocardiogram LV: left ventricular

Notably, the admission troponin was 0.06 ng/mL or greater in patients with ventricular dysfunction by echocardiogram (Figure 3, Table 5).

**Table 5:** Characteristics of patients with ventricular dysfunction AML: acute myeloid leukemia, ARVC: arrhythmogenic right ventricular cardiomyopathy, BNP: B-type natriuretic peptide, cMRI: cardiac magnetic resonance imaging, ECG: electrocardiogram, ECMO: extracorporeal membranous oxygenation, EF: ejection fraction, LAD: left axis deviation, LGE: late gadolinium enhancement, LOS: length of stay, LV: left ventricle, OSA: obstructive sleep apnea, RAD: right axis deviation, RV: right ventricle, T2DM: type 2 diabetes mellitus, VV: venovenous

	Age, gender	Medical history	Cardiac history	Symptoms	ECG	Troponin on admission, peak (ng/mL)	BNP on admission (pg/nL)	Echo EF, other	cMRI	LOS (days)	Outcome
A	5 months, male	None	None	Unresponsive at home	Low voltage	3.24, 3.24	-	EF: 44%	-	32	Death (hypoxic respiratory failure)
B	14.5 years, female	AML, T2DM, Friedreich ataxia, OSA, hypothyroid	ECG: T-wave inversions, echo: mild LV hypertrophy, normal function	Chest pain, shortness of breath	RAD	1.15, 1.15	88	EF: 50%, symmetric LV hypertrophy	Normal	8	Normalized function on follow-up
C	16.5 years, female	Pregnancy (at 25 weeks)	None	Chest pain, fever	ST segment abnormal	0.43, 0.43	245	EF: 50%, LV basilar segment hypokinesis	-	13	Delivery via c-section
D	18 years, male	Asthma	None	Chest pain, emesis, diarrhea	LAD, low voltage, ST segment elevation	4.86, 310	-	EF: 41%, dilated RV	Dilated RV, LGE	11	ARVC diagnosis
E	18 years, male	Autism, morbid obesity	None	Chest pain, fever, emesis	Normal	0.060, 0.060	-	EF: 49%, dilated RV	-	50	VV ECMO x23d, post-ECMO course without complication

**Figure 4:**
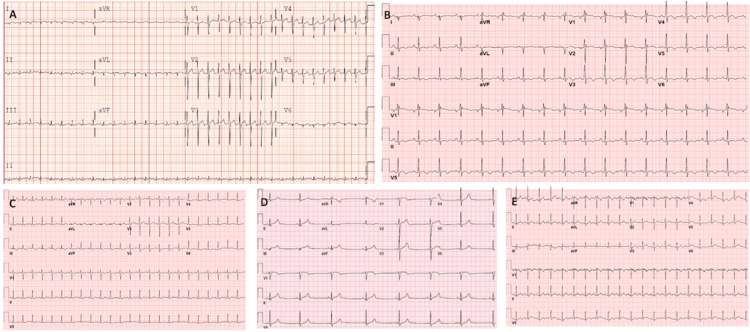
Electrocardiograms of patients with ventricular dysfunction

Of patients with ventricular dysfunction, four (80%) had abnormal electrocardiograms (Figure 4). Of the two cardiac MRIs performed, one was normal, while the other showed findings suggestive of arrhythmogenic right ventricular cardiomyopathy. No patients had arrhythmias.

Patterns in cardiac abnormalities

There were no differences in age, self-reported gender, race, ethnicity, or medically disadvantaged status between the patient groups with and without cardiac abnormalities. The proportion of patients with cardiac abnormalities did not significantly change over the study period (r = 0.004, p = 0.938) (Figure 2).

Patients with cardiac abnormalities were more likely to exhibit chest pain or elevated C-reactive protein, require any antiviral medication (especially hydroxychloroquine) or steroids, and require admission to intensive care, respiratory support, or a vasoactive medication (Table 6).

**Table 6:** Patient characteristics and incidence of cardiac abnormalities

Characteristic	Cardiac abnormality (n = 58)	No abnormality (n = 73)	p
Age, years (interquartile range)	17 (6-19)	15 (2-19)	0.89
Area deprivation index, decile (interquartile range)	3 (2-5)	3 (2-5)	0.39
Existing medical condition			
Any	40 (69%)	36 (49%)	0.032
Congenital heart disease	4 (7%)	4 (6%)	0.73
Asthma	13 (22%)	9 (12%)	0.16
Obesity	11 (19%)	12 (16%)	0.82
Symptoms on presentation			
Any	50 (86%)	55 (76%)	0.13
Fever	31 (53%)	37 (51%)	0.86
Cough	20 (35%)	26 (36%)	>0.99
Shortness of breath	16 (28%)	10 (14%)	0.076
Chest pain	10 (17%)	2 (3%)	0.005
Duration of illness, days (interquartile range)	2 (1-6)	3 (1-7)	0.78
Testing abnormality			
Complete blood count	38 (66%)	49 (67%)	0.86
Inflammatory marker	46 (87%)	43 (68%)	0.027
C-reactive protein	42 (89%)	35 (70%)	0.024
Procalcitonin	18 (31%)	15 (40%)	0.15
Ferritin	27 (61%)	14 (39%)	0.072
Coagulation markers	31 (65%)	26 (46%)	0.077
D-dimer	26 (65%)	22 (60%)	0.665
Fibrinogen	14 (61%)	12 (55%)	0.77
Chemistries	33 (59%)	38 (53%)	0.59
Hypokalemia	9 (16%)	7 (10%)	0.42
Hypocalcemia	18 (31%)	12 (17%)	0.058
Hypomagnesemia	5 (10%)	1 (2%)	0.20
Abnormal chest radiograph	35 (60%)	25 (45%)	0.034
Intensive care admission	28 (48%)	22 (30%)	0.020
Medication administration			
Antiviral	26 (45%)	14 (19%)	0.0021
Remdesivir	10 (17%)	7 (10%)	0.30
Hydroxychloroquine	19 (33%)	8 (11%)	0.0041
Azithromycin	10 (17%)	7 (10%)	0.30
Anti-inflammatory	17 (29%)	7 (10%)	0.0057
Steroid	13 (22%)	3 (4%)	0.0023
Tocilizumab	5 (9%)	4 (6%)	0.51
Antibiotic	29 (40%)	34 (47%)	0.73
Vasoactive medication	8 (14%)	2 (3%)	0.022
Respiratory support			
Any	31 (54%)	22 (30%)	0.0078
Mechanical ventilation	13 (22%)	8 (11%)	0.095
Length of stay, days (interquartile range)	6 (3-15)	3 (1-8)	0.021

Eight patients with underlying congenital or pediatric heart disease were included in our cohort (Table 7). New cardiac abnormalities were identified in four of these eight patients, which included pericardial effusion, elevated troponin or B-type natriuretic peptide, and electrocardiogram changes. None of these patients developed ventricular dysfunction.

**Table 7:** Characteristics of patients with pediatric heart disease AV: atrioventricular, BRUE: brief resolved unexplained event, ECG: electrocardiogram, EP: electrophysiology, TAPVR: total anomalous pulmonary venous return, TOF: tetralogy of Fallot, URI: upper respiratory infection, VSD: ventricular septal defect, VT: ventricular tachycardia

	Age, gender	Cardiac diagnosis	Symptoms	New cardiac abnormality?	ECG change	Echocardiogram change	Admission reason
A	2 months, male	VSD (unrepaired)	Cough, emesis, irritability	Yes (ECG change)	Repolarization abnormalities; prolonged QTc	None	BRUE
B	3 months, male	TAPVR (repaired)	Fever, cough, dyspnea	No	None	None	Viral URI
C	3 years, male	VSD (unrepaired)	Emesis, cough, dyspnea	Yes (pericarditis)	ST segment elevation	Pericardial effusion	Pneumonia
D	4 years, female	Patent ductus arteriosus (unrepaired)	Fever	No	None	None	Osteomyelitis
E	7 years, male	Complete AV septal defect (repaired)	Fever, cough, dyspnea	Yes (elevated BNP: 106 pg/mL (106 ng/L))	None	None	Acute respiratory distress syndrome
F	12 years, female	Double outlet right ventricle (repaired)	Fever, emesis, diarrhea	Yes (elevated troponin: 0.10 ng/mL (0.10 µg/L))	None	None	Acute respiratory distress syndrome
G	16 years, female	Incessant VT (following EP study with ablation)	Abdominal pain	No	None	None	Pyelonephritis
H	18 years, female	TOF with complete AV septal defect (repaired)	Fever, cough	No	None	None	Pneumonia

Three patients in our cohort required venovenous extracorporeal membrane oxygenation for refractory hypoxemia. One patient (Table 5, patient E) had an elevated troponin at 0.06 ng/mL and mildly diminished left ventricular function by echocardiogram, with an ejection fraction of 49%, with a normal electrocardiogram. The two remaining patients had normal cardiac testing. All patients were successfully decannulated and were ultimately discharged home. No patients required venoarterial extracorporeal membrane oxygenation.

Outcomes and discharge

Testing for cardiac abnormalities was repeated prior to discharge in 30 of the 56 (54%) patients with cardiac abnormalities. Sixteen (28%) of these patients had electrocardiogram abnormalities on discharge, including 13 patients with persistent repolarization abnormalities, two with prolonged corrected QT interval, two with left atrial enlargement, and two with axis deviation. Echocardiogram, troponin, and B-type natriuretic peptide normalized or significantly improved in all patients prior to discharge.

Three patients in our population died during hospitalization. One patient (patient A, Table 5) was a five-month-old with left ventricular dysfunction who was found unconscious and without a pulse after a nap despite having no prior symptoms of COVID-19. This child was also found to have parainfluenza virus. He suffered a significant neurologic injury, required prolonged intubation, and ultimately succumbed to worsening respiratory failure. The second patient, a six-year-old male, presented following drowning, suffering an irreversible neurologic injury, having previously shown no symptoms of COVID-19. This patient had a prolonged corrected QT interval (470 milliseconds), although no additional cardiac testing was performed. The third patient, a nine-year-old female, presented with altered mental status following the rupture of an arteriovenous malformation, suffering an irreversible neurologic injury, having shown no symptoms of COVID-19. This patient had an elevated high-sensitivity troponin (17,212 ng/L) and B-type natriuretic peptide (269 pg/mL) on admission, although no electrocardiogram or echocardiogram was obtained.

Follow-up

Outpatient follow-up data were available for 23 of 56 (41%) patients with cardiac abnormalities. The median follow-time up was 11 weeks from discharge (interquartile range: 5-24 weeks). Of the 23 patients with repeat electrocardiograms, eight (35%) still had an abnormal test. Specifically, two of the four surviving patients with ventricular dysfunction had persistent repolarization abnormalities by electrocardiogram. Echocardiographic abnormalities normalized in all patients. Troponin and B-type natriuretic peptides were not repeated on an outpatient basis.

## Discussion

Data on cardiac abnormalities during acute COVID-19 infection in the pediatric population remain lacking compared to adult studies. Current literature suggests that most children do well, although mechanical ventilation, vasoactive medications, or extracorporeal membrane oxygenation have been required albeit rarely, with deaths reported [19]. Patients who are sicker, with more preexisting conditions or more severe symptoms on presentation, have worse outcomes [19]. However, few studies have looked primarily at the effects of acute COVID-19 on the heart in children. Here, we report the largest US cohort to date of hospitalized children and adolescents with cardiac abnormalities in acute COVID-19. Of the children in our cohort admitted due to COVID-19 and who underwent cardiac testing, nearly half had an abnormal result. Despite this finding, only a small number of patients had significant abnormalities such as ventricular dysfunction, with nonspecific electrocardiogram findings accounting for many of the detected abnormalities.

We identified five patients with ventricular dysfunction: four adolescents whose ventricular functions recovered and one infant who ultimately died from reasons likely other than COVID-19. Except for the infant, these patients had both repolarization abnormalities and elevated troponin greater than 0.04 ng/mL (0.04 µg/L). Other factors may be useful in identifying which children are at increased risk of cardiac involvement. For example, patients with cardiac abnormalities were more likely to present with chest pain or elevated inflammatory markers. These patients also were generally sicker, being more likely to require intensive care admission, antiviral medications, steroids, vasoactive medications, or respiratory support.

We reported two patients with ventricular dysfunction whose cardiac abnormality might not be directly attributable to COVID-19. One infant (patient A, Table 5) appeared to suffer a sudden event while sleeping without prior viral symptoms, was found unresponsive, and suffered a severe hypoxic injury with ventricular dysfunction. This clinical picture is more consistent with sudden infant death syndrome than COVID-19. One adolescent (patient D, Table 5) presented with symptoms initially consistent with COVID-19-related myocarditis but with significant right ventricular involvement. With follow-up, this patient was diagnosed with arrhythmogenic right ventricular cardiomyopathy, which was likely unmasked by his viral illness. Despite the likelihood that the cardiac abnormalities seen in these two patients were caused by etiologies other than COVID-19, we also could not definitively exclude a link between COVID-19 and their underlying disease. Thus, they were included in our cohort for completeness.

Patients with congenital heart disease were not at increased risk of developing cardiac abnormalities in our cohort, and none of the patients with ventricular dysfunction had underlying congenital heart disease. However, the small number of patients with congenital heart disease limited our ability to make inferences. This is similar to existing literature [20].

The use of antivirals was associated with cardiac abnormalities in our cohort. Hydroxychloroquine use in particular was associated with cardiac abnormalities, while patients with cardiac abnormalities were not more likely to have received remdesivir or azithromycin. Both hydroxychloroquine and azithromycin are known to prolong the corrected QT interval, possibly explaining the association between hydroxychloroquine and electrocardiogram changes observed in our study [21]. An alternative explanation is that patients who were sick enough to require antivirals were sick enough to be at increased risk of cardiac abnormalities. The use of respiratory support, an indication for antiviral therapy in COVID-19 patients, was independently associated with cardiac abnormalities.

Although heart block has previously been reported to occur with COVID-19, no patient in our cohort developed this condition [10,22]. The etiology of heart block in patients with COVID-19 is still uncertain, further confounding efforts to determine the reason this complication was not observed in our cohort [23]. Unlike prior studies, our cohort only included patients with acute COVID-19 and not MIS-C, which may explain this finding.

The risk factors for overall poor outcomes in COVID-19 are known to include lower socioeconomic status [8,24]. Our study did not identify age, gender, race, ethnicity, or medically disadvantaged status as risk factors for cardiac abnormalities. Existing literature also describes hypertension, asthma, obesity, and diabetes mellitus as risk factors for cardiac injury in adults [24,25]. We did not find an association between cardiac abnormality and any one specific condition, further suggesting that the link between comorbid conditions and severe COVID-19 disease in children is not as clear as the link in adults [9].

The inclusion of longitudinal follow-up data proved difficult to obtain via retrospective chart review, as we were only able to collect this data on about one-third of the patients with cardiac abnormalities. Patient noncompliance or follow-up with other centers may account for a portion of these missing patients. Similar to previously reported studies, all patients with ventricular dysfunction had improved function at the time of follow-up [26,27]. However, some patients demonstrated persistence of electrocardiogram changes, with only 71% of the patients seen as an outpatient having improvement in their cardiac testing. This is important, as the long-term consequences of COVID-19 infections, especially in patients with cardiac abnormalities, are still unknown. Although our results are limited, they support the current expert opinion that all patients with at least moderate symptoms of COVID-19 undergo a cardiac evaluation prior to engaging in sports [28].

Similar studies have identified the prevalence of cardiac injury in COVID-19 to be about 10%-20% in both children and adults [2]. While our study identified a cardiac abnormality in close to half of the tested patients, we identified an elevated troponin in 18% of patients. This is in contrast to other studies reporting an incidence of elevated troponin to be closer to 1% of patients [1,5]. Selection bias may account for a portion of this discrepancy, as not every admitted patient underwent cardiac testing. However, similar to existing literature, we found elevated inflammatory markers and intensive care admission to be risk factors for cardiac injury [7].

Strengths and limitations

The strengths of our study include its multicenter nature, allowing for one of the largest samples, thus far assessing the cardiac effects of COVID-19. Despite its multicenter nature, our study is still underpowered to identify small effects (e.g., the effect of existing medical conditions on cardiac abnormalities) or more specific details (e.g., the effect of specific antiviral agents on cardiac abnormalities). An analysis of the characteristics, symptoms, or test results that would best predict ventricular dysfunction would be especially useful in guiding the evaluation of patients with COVID-19, although this would require a significantly larger sample size.

This study is also limited by its retrospective observational nature. Decisions regarding screening, testing, and follow-up were made by the medical team at the time of patient care, thus leaving some data points incomplete. In addition, electrocardiogram and echocardiogram findings were subject to the interpretation of the clinical team and were not reinterpreted by the investigators. While this prevents standardization of findings such as abnormalities of the ST segment or T wave, there are no universally agreed-upon definitions for these terms, and deferring to the judgment of the clinical teams best represents how these findings are utilized in practice.

By only including admitted patients in our cohort, we may have omitted some patients with cardiac abnormalities, although our data would suggest that patients who are not sick enough to require admission are less likely to have cardiac abnormalities. Additionally, patient management of this novel pandemic evolved over the study period. Although the ratio of patients tested for cardiac abnormalities did not change over the course of the study period, the medications changed dramatically, subjecting patients to different adverse effects at different time points. Finally, the virus has evolved over the course of the study period, modifying its clinical impact [29]. Through this observational study, we were unable to control for such variation in time.

## Conclusions

In summary, less than half of pediatric patients admitted with COVID-19 had cardiac abnormalities, although most of these abnormalities were electrocardiogram findings, with very few patients having ventricular dysfunction. Patients who had existing medical conditions, as well as those who were sicker at presentation, were more likely to have cardiac abnormalities. The combination of elevated troponin and repolarization abnormalities on electrocardiogram may portend ventricular dysfunction in affected patients.

Our study suggests that an electrocardiogram and troponin have a high utility for identifying children with COVID-19 who have cardiac abnormalities or ventricular dysfunction. Given the low cost and availability of these tests, we recommend that these two tests be obtained in all hospitalized patients with COVID-19, especially those who are symptomatic or with preexisting conditions. Adjunctive evaluation such as inflammatory markers or chest radiographs may be helpful but are likely not necessary to exclude cardiac disease. Obtaining an echocardiogram may be required only in admitted patients with repolarization abnormalities by electrocardiogram or in patients with an elevated troponin.
